# The Proper Strength of Antiseptic Solutions to Be Used in Surgery

**Published:** 1882-07

**Authors:** 


### THE PROPER STRENGTH OF ANTISEPTIC SOLUTIONS TO BE USED IN SURGERY.

Dr. Edmund Andrews has recently made some experiments (*Chic. Med. Journ. and Exam.*) on the value of thymol as an antiseptic in surgery. As a result of his observations, he concludes that it is of very little value. In this conclusion he is supported by a recent report of the Imperial Board of Health of Germany. Incidentally Dr. Andrews discusses the proper strength of carbolic acid solutions to be used in the treatment of abscess cavities, and relates the following case :

"In a recent case which fell under my advice, a lumbar abscess was held in perfect subjection for months by forced injections of one part of carbolic acid to forty of water. Circumstances having placed the patient temporarily under the care of a practitioner who had faith in weak solutions, septic action was promptly set up, and in two weeks the man was in the full enjoyment of hectic fever and of a putrid discharge from the abscess. Fortunately he returned in time within my reach, and a few days of forced injection of carbolic acid, at first in the proportion of one part to thirty and afterward one to forty, arrested the mischief both locally and constitutionally, and restored the patient to comfort and safety."

